# Health-care burden of metabolic dysfunction-associated steatotic liver disease in European tertiary-care outpatient clinics (CONSTANS): a prospective multicentre cohort study

**DOI:** 10.1016/j.lanepe.2026.101893

**Published:** 2026-10-29

**Authors:** Vlad Ratziu, Jean-François Dufour, Maharajah Ponnaiah, Raluca Pais, Maximilian Thormann, Elisabetta Bugianesi, Fabio Marra, Marie Boyle, Kate Hallsworth, Silvia Aspite, Sylvia Drazilova, Federico Ravaioli, Manuel Romero Gomez, Leila Kara, Quentin M. Anstee, Jörn Schattenberg

**Affiliations:** a Sorbonne Université, Paris, France; b ICAN Institute for Cardiometabolism and Nutrition, Paris, France; c Assistance Publique-Hôpitaux de Paris, Hepatogastroenterology Department, Pitié-Salpêtrière Hospital, Paris, France; d INSERM UMRS 1138 CRC, Paris, France; e Inselspital, Universitätsspital Bern, Bern, Switzerland; f Division of Internal Medicine, Hepatobiliary and Immunoallergic Diseases, IRCCS Azienda Ospedaliero-Universitaria di Bologna, Italy; g Department of Clinical and Experimental Medicine, University of Florence, Florence, Italy; h Translational & Clinical Research Institute, Faculty of Medical Sciences, Newcastle University, Newcastle upon Tyne, UK; i 2nd Department of Internal Medicine, PJ Safarik University, Faculty of Medicine and L Pasteur University Hospital, 04011, Kosice, Slovakia; j Department of Internal Medicine, Hospital Poprad, Slovakia; k Hospital Universitario Virgen del Rocío, Instituto de Biomedicina de Sevilla, Sevilla, Spain; l Newcastle NIHR Biomedical Research Centre, Newcastle upon Tyne Hospitals NHS Trust, Newcastle upon Tyne, UK; m Department of Medicine, University Medical Centre Mainz, Germany

**Keywords:** Metabolic-dysfunction associated steatotic liver disease, Fibrosis, Health-care burden, Non-invasive tests, Cost

## Abstract

**Background:**

Despite modeling-based epidemiological studies of MASLD, precise estimates of the prevalence and health-care resource utilization in outpatient clinical practice are unknown. We assessed, in a real-life setting, the prevalence of suspected/confirmed MASLD and described health-care resource utilization.

**Methods:**

A prospective, observational study of patients attending the outpatient hepato-gastroenterology clinics of eight European tertiary care centers during three months. Follow-up data of patients with suspected/confirmed MASLD were recorded 12 months after the screening visit: final diagnosis, disease stage, diagnostic work-up and the use of health-care resources.

**Findings:**

5240 outpatients from Germany, France, United Kingdom, Switzerland, Italy, Spain and Slovakia were screened. The main reasons for visit were: chronic viral hepatitis, 33.4% (1749/5240), miscellaneous hepatobiliary conditions including neoplasms, 32.9% (1725/5240) and known/suspected MASLD, 24.7% (1293/5240) (pooled prevalence of 26.1% (95% CI: 19.1–34.7)). 1134 suspected/confirmed MASLD patients were included, the diagnosis was confirmed in 80.2% (909/1134). Significant fibrosis was documented in 22.7% (257/1134) and cirrhosis (including burn-out MASLD) in 21.3% (241/1134). Procedures performed differed between countries, overall 66.4% (753/1134) had imaging studies, 65.3% (740/1134) non-invasive fibrosis tests, 20.4% (231/1134) liver biopsy, while referral for further cardiometabolic assessment or therapy was minimal (144/1134, 15.8%). The cost of all procedures and visits was in the range of that recommended for exploring hepatitis C before treatment, although highly variable between countries.

**Interpretation:**

MASLD was the second most frequent single cause for outpatient visits in eight European tertiary care centers. Significant fibrosis including cirrhosis was diagnosed in 44% of patients. The cost of required additional visits and procedures was equivalent to that recommended for hepatitis C before antiviral treatment.

**Funding:**

IMI2-Grant Agreement 777377-LITMUS.

**Research in context**

**Evidence before this study**

Metabolic-dysfunction associated steatotic liver disease (MASLD) is the most common chronic liver disease in Western countries, driven by the increasing prevalence of obesity and type 2 diabetes. Although only a minority of patients develop complications requiring hospitalization for decompensated cirrhosis, hepatocellular carcinoma, or liver transplantation, most patients are managed in outpatient settings. Current guidelines recommend referral of individuals with metabolic risk factors for case-finding of advanced liver disease. However, the healthcare burden of MASLD in real-world specialized outpatient clinics has not been well quantified.

We searched PubMed, using the terms “MASLD”, “MASH”, “MAFLD”, “NAFLD”, “NASH”, combined with “real-world data”, “outpatient clinic”, “healthcare resources”, “healthcare burden”, “diagnostic procedures”, and “cost analysis” between Nov 21st 2025 and January 5th 2026. We identified retrospective surveys in selected populations, registry-based studies of hospitalized patients, and modeling studies based on obesity or diabetes prevalence. We found no prospective, multicentre studies assessing the healthcare burden of MASLD in specialized outpatient clinics using adjudicated patient-level data, nor studies evaluating MASLD-related visits in the context of overall hepatobiliary practice.

**Added value of this study**

This prospective, multicentre, multinational real-world study conducted in specialized outpatient clinics from seven European countries provides adjudicated patient-level data on the prevalence of MASLD-related visits among all hepatobiliary consultations. It quantifies diagnostic and therapeutic procedures, associated costs, and disease severity, and allows comparison of healthcare resource utilization across countries.

**Implications of all the available evidence**

Prospectively collected, adjudicated data from routine clinical practice provide a robust estimate of the healthcare burden and resource utilization associated with MASLD in tertiary care settings. These findings may inform evidence-based healthcare planning and resource allocation for this prevalent liver disease.

## Introduction

With the rise of the prevalence of obesity and diabetes over the past few decades metabolic-dysfunction associated steatotic liver disease (MASLD) has become the most common chronic liver disease worldwide.^1^ MASLD is a largely asymptomatic disease, usually diagnosed based on the presence of excess liver fat by imaging or of altered liver enzymes in patients with metabolic risk factors.^2^ Suspicion of MASLD requires confirmation and staging of the disease, almost always performed in specialized hepatological settings. Since liver biopsy is invasive, there are numerous non-invasive procedures to confirm and stage the disease are being used, which results in divergent patterns of practice across specialized clinics.^3^ Regional or national specifics of the epidemiology of chronic liver diseases may also influence the interest and completeness of the exploration of suspected or diagnosed MASLD in different population areas. All these elements dictate whether exploration of MASLD currently occupies a central or a marginal place in specialized hepatology units. While a fraction of MASLD patients will develop cirrhosis, hepatocellular carcinoma or end-stage liver disease,^4^ most patients are managed in outpatient settings, both for the initial exploration and disease follow-up. Despite this, data on the actual health-care burden of MASLD in a real-world, outpatient setting is very limited. Data from Europe are scarce and rely on modeling based on the prevalence of obesity or diabetes,^5^^,^^6^ registry-based studies^7^^,^^8^ or select cases from physician surveys^9^ without distinction between the few severe cases requiring hospitalization, intense work-up and higher costs and the majority of milder or asymptomatic forms of the disease. Available studies do not assess the burden attributable to MASLD in the context of chronic liver diseases seen in outpatient clinics, and, most importantly, do not rely on actual patient-level data processed and adjudicated by specialized hepatogastroenterologists. Given current estimates of the future incidence of MASLD^10^ and recent recommendations for case-finding and referrals for hepatological exploration of patients with metabolic risk factors,^11^, ^12^, ^13^, ^14^ it is urgent to collect real-world, direct, exhaustive and precisely adjudicated patient-level data on the burden of MASLD in outpatient specialized clinics. These data could help determine the real burden of the disease and hence anticipate a rational use of medical and human resources for managing this increasing patient population.

We therefore performed a prospective study in tertiary care hepatological centers from seven European countries in order to analyze data from patients seen in an outpatient setting. The objectives were to assess the prevalence of a suspected and confirmed diagnosis of MASLD, its fibrotic severity, the procedures actually performed and the cost related to the overall management and monitoring of these patients.

## Methods

CONSTANS (Consultations pour stéatohépatites ambulatoires en milieu hospitalier) is a multicentric study performed between Sept 3rd 2018 and January 16th 2020 in eight centers: Pitié-Salpêtrière Hospital, Paris, France; University Medical center of the Johannes Guttenberg University, Mainz Germany; Inselspital, Bern, Switzerland; Freeman Hospital, Newcastle United Kingdom; Department of Medical Sciences, University of Torino, Torino Italy; Hepatogastroenterology Unit, University of Florence, Florence, Italy; Digestive Diseases Unit and CIBERhed, Virgen Del Rocío University Hospital, Sevilla, Spain; and the Department of Internal Medicine, Poprad Hospital, Poprad, Slovakia. All patients consulting a participating physician (PP) for a hepatological reason, at the outpatient clinic of any of these hospitals during a 3-month period, were prospectively included (baseline visit). Participating physicians were board-certified hepatogastroenterologists with a hepatological practice of at least 20%; physicians from the same institutions with a predominant gastroenterological or gastro-oncology practice (other than liver and biliary cancers) and with less than 20% of their patients usually consulting for a hepatological reason, were not asked to participate. Patients were included whether this was their first or a follow-up visit. Patients consulting primarily for a gastrointestinal condition or complaint (including irritable bowel syndrome) or for a gastrointestinal, non-hepatic, malignancy were not included. A schematic representation of the study design is shown in Supplementary Figure S1.

The main reason for the baseline visit was determined by the PP after taking into account all clinical and biological parameters at their disposal during the baseline visit. The reason was categorized as follows: a) viral hepatitis (acute or chronic); b) alcohol-related liver disease (including alcohol use disorder or at risk alcohol consumption, i.e. >50 g alcohol/day); c) known (i.e. already diagnosed) MASLD; d) abnormal liver enzymes; e) suspicion of MASLD defined as either steatosis detected by imaging or abnormal liver enzymes together with metabolic risk factors [(overweight (BMI >25 kg/m^2^) or visceral adiposity (waist circumference >88 cm women, 100 cm men), type 2 diabetes, arterial hypertension, dyslipidemia)]; f) exploration of increased serum ferritin; g) cryptogenic cirrhosis; and h) miscellaneous hepatobiliary disorders (including autoimmune, genetic vascular liver disorders, drug-induced liver disease, known hepatic or biliary tumors, work-up for a hepatic nodule). Alcohol consumption was determined by self-reporting from the patient and a review of available medical history data, if available.

### Patients

Only patients belonging to the categories “known MASLD”, “altered liver enzymes”, “suspicion of MASLD”, “increased serum ferritin” or “cryptogenic cirrhosis” were further considered for the current study. Their proportion among the total number of patients was calculated. Patients belonging to the following categories: viral hepatitis, alcohol-related liver disease (including alcohol-related cirrhosis), autoimmune hepatic or biliary diseases, genetic diseases (e.g. hemochromatosis, Wilson), suspicion of drug-induced liver injury, hepatic tumors, or miscellaneous diseases (vascular diseases, follow-up of organ transplant patients etc) were not further investigated for the purpose of this study. For each patient the PP indicated the decision taken at the end of the baseline visit which could be one or several of the following: scheduling of a follow-up visit; prescription of blood tests; prescription of imaging procedures (ultrasound, CT scan, MRI etc); of non-invasive fibrosis tests (elastography or serum biomarkers) or liver biopsy.

One year after the baseline visit the medical records were reviewed for the number of follow-up visit(s) and for procedures actually performed. The results of the performed procedures were reviewed in order to determine: 1) the final diagnosis: confirmed MASLD, indeterminate or other; 2) the severity of liver injury. The adjudication for the final diagnosis and disease fibrotic severity was done by the participating physician, was monitored centrally for internal consistency (LK) and underwent final confirmation based on the totality of the individual patient data by one of the authors (VR).

### Cost analysis

The cost analysis was performed for each individual country given the inter-national differences in health care systems and cost of medical procedures. The participating hospitals were interrogated for the estimated cost of specialized medical visits, imaging diagnostic procedures (ultrasound, CT-scan, MRI, elastography), liver biopsy (as an outpatient procedure), blood tests (including liver enzymes, lipid panel, glycemia, fasting insulin). The cost incurred by the total number of medical visits, blood tests, imaging procedures, liver biopsies in each participating center was calculated for patients who actually had these procedures performed (since not all patients had all procedures performed). The total cost per patient included in each country was then calculated by dividing the total cost estimated for each center by the number of patients included in that center. This total cost was then compared to the cost of investigating and treating a patient referred for HCV infection. The cost for the initial visit of a patient referred for a diagnosis of (suspected) HCV infection included: the medical visit, blood tests (liver enzymes), HCV viral load, HCV genotype, abdominal ultrasound, elastography (vibration controlled transient elastography). Since universal treatment is currently recommended for HCV elimination, an additional cost generated by the decision to treat and monitor antiviral response was calculated and included two additional visits and two additional viral load measurements (end of treatment and 12-week post treatment). The overall cost per included patient for a MASLD or suspected MASLD referral was then compared, for each individual center, with the overall cost before the decision to treat and after HCV antiviral treatment (cost of medication excluded) for a patient referred for HCV infection.

### Statistical analysis

As this was an observational study, no formal sample size was calculated although we estimated a prevalence of MASLD among outpatient visits at around 25%. Analyses were conducted using the adjudicated follow-up cohort (n = 1134). Continuous variables are summarized as mean (standard deviation) or median (interquartile range), and categorical variables as counts (percentages).

Between-center variability was assessed through random-effects meta-analyses of proportions at the hospital level. Center-specific prevalence estimates were calculated, and pooled estimates were reported with 95% confidence intervals (CI). Statistical heterogeneity was quantified using the I^2^ statistic and τ^2^. Associations between patient characteristics and MASLD-related diagnosis, severe liver disease, procedure burden, and cost were assessed using multivariable regression models appropriate to outcome type. Logistic regression models were used for binary outcomes, Poisson regression models for procedure counts, and log-transformed linear regression models for cost analyses. Results are presented as odds ratios or cost ratios with 95% confidence intervals. Covariates included age, sex, body mass index (BMI), diabetes, arterial hypertension, dyslipidaemia, and patient status (new patient vs follow-up). Missing data were quantified for all variables. The primary analyses used a complete-case approach; sensitivity analyses were performed by restricting the cohort to new patients only.

Missing data were handled using a complete-case approach in the primary analyses. To assess the robustness of the findings, sensitivity analyses using multiple imputation by chained equations (MICE) were performed. Imputation models included all covariates used in the regression analyses together with the corresponding outcome variable, in accordance with current recommendations. Twenty imputed datasets were generated and combined using Rubin's rules.

### Ethical approval

The study was approved by the Ethics Committee CCTIRS (Comité Consultatif sur le Traitement de l’Information en matière de Recherche dans le domaine de la Santé) under the reference Ref: 16.807.

### Role of the funding source

The study received funding from the Innovative Medicines Initiative 2 (IMI2) Joint Undertaking under Grant Agreement 777,377 (LITMUS Consortium). The funder had no role in study design, data collection, data analysis, interpretation or writing of the report.

## Results

### Prevalence of a MASLD-related reason for consultation

The study included 5240 patients seen at the baseline visit: 1973 in Germany, 914 in Switzerland, 848 in France, 694 in the UK, 470 in Italy, 191 in Spain and 150 in Slovakia. The study flow-chart is shown in Fig. 1. Mean age was 54.74 years (s.d. 14.95), 53% were males. Reasons for the visit were: chronic viral hepatitis 33.4%, alcohol-related liver disease/excessive alcohol consumption 9.0%, known MASLD 11.8%, altered liver enzymes or ferritin 8.1%, steatosis on imaging studies 2.5%, cryptogenic cirrhosis 2.4%, other hepatobiliary conditions including hepatobiliary neoplasms 32.9% (Fig. 2). Overall, initial visits represented 21% and the rest were follow-up visits of patients already seen previously by the PP in the same facility. Only 4.3% of patients were included in a therapeutic protocol. Based on the reason for the visit established by the PP during the baseline visit, the proportion of patients consulting for a MASLD-related diagnosis (either known MASLD, suspected MASLD or cryptogenic cirrhosis) was 24.7% (1293/5240, 99% CI 23.2%–26.2%, p = 0.33 vs null hypothesis of a prevalence of 25%). This proportion ranged from 15% in Germany to 43% in Slovakia (Supplementary Figure S2). Fig. 2b shows the result of a random-effects meta-analysis of proportions to estimate the prevalence of known or suspected MASLD among all reasons for outpatient visits taking into account centre heterogeneity including sample size, differences in case mix and referral patterns. The pooled prevalence was 26.1% (95% CI: 19.1–34.7), with substantial heterogeneity across centers (I^2^ = 97.1%). These 1293 patients were further considered for the follow-up study.

**Fig. 1:**
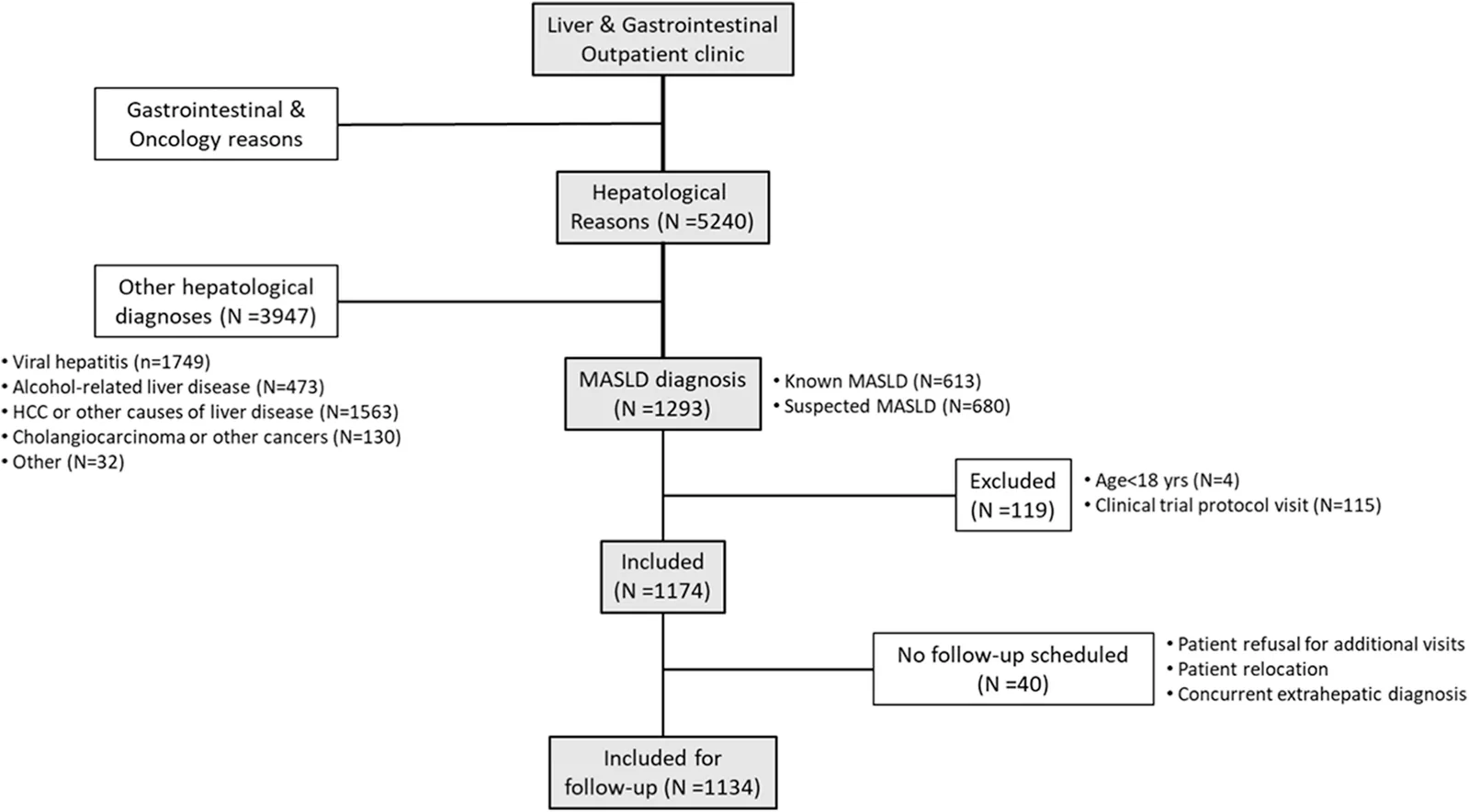
Study flow-chart.

**Fig. 2:**
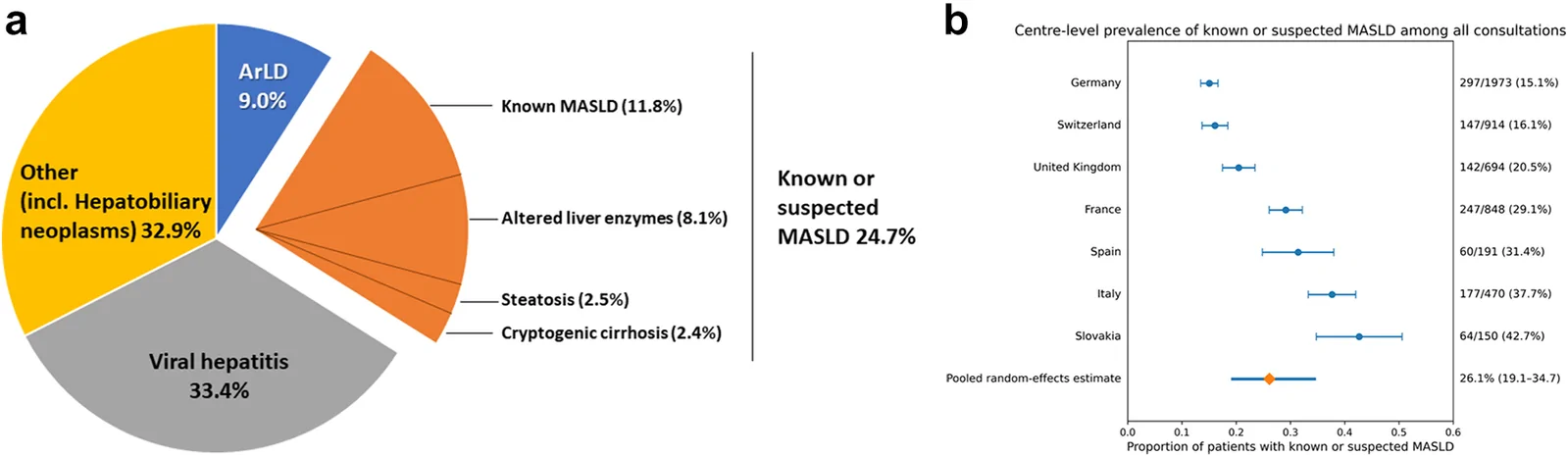
**a)** Crude proportion of reasons for initial outpatient visit in all centers combined; **b)** pooled estimate by meta-analysis using a random-effects model accounting for between-centre variability (heterogeneity I^2^ = 97.1%, τ^2^ = 0.289).

Of these 1293 patients, 119 were excluded from follow-up visits or procedures because of age <18 years, participation in a MASH therapeutic protocol that imposed specific follow-up procedures or because of other reasons. Out of the remaining 1174 patients, 40 were not scheduled for a follow-up visit or for other investigations (Fig. 1). Thus, 1134 patients were included for follow-up procedures: 297 from Germany, 247 from France, 177 from Italy, 147 from Switzerland, 142 from the UK, 64 from Slovakia and 60 from Spain. Mean age was 56.9 years (s.d. 13.9) and 53% were males (Supplementary Table S1). Among these 1134 patients, new cases (i.e. seen for the first time in the corresponding clinic) accounted for 37% (N = 419), while 63% (N = 715) have already been seen before by the PP. The main reason for the medical visit in these 1134 patients was: known MASLD 45.9% (N = 521), increased liver enzymes 30.6% (N = 347), increased serum ferritin 2.4% (N = 27), steatosis on imaging studies 10.8% (N = 123) and cryptogenic cirrhosis 10.2% (N = 116).

### Prescribed follow-up

Among the 1134 patients with a follow-up visit scheduled, 95% had one or several procedures planned after the initial visit: blood tests in 89% of patients, an imaging procedure in 59.5%, elastography in 41%, serum fibrosis markers in 21.5% and a liver biopsy in 14%. Among imaging procedures, the most frequently prescribed was abdominal ultrasound (92%). When comparing patients with known MASLD (N = 521) with those with suspected MASLD (N = 497, which includes patients consulting for increased liver enzymes, increased serum ferritin or steatosis on imaging) there were no notable differences in scheduled procedures, except, in the former group, a lower rate of imaging studies (48% vs 68%, respectively) and liver biopsy (12.5% vs 17%, respectively) (Table 1). Liver biopsy was ordered more commonly in new patients than in follow-up patients (17.7% of them vs 12.3%, respectively). Among participating countries the prescription for liver biopsy was largely variable as it ranged from 40% in Italy and 20.5% in Germany to 0% in Slovakia. Liver biopsy was ordered more often in cases of suspected MASLD (53% of biopsies) than of known MASLD (40% of biopsies) and only marginally in those with cryptogenic cirrhosis (7% of biopsies). In contrast, elastography or proprietary fibrosis markers were performed equally in those with suspected and known MASLD.

**Table 1:** Diagnostic procedures scheduled in patients suspected MASLD (N = 497), known MASLD (N = 521) or cryptogenic cirrhosis during the first (initial, baseline) visit.

Procedure	All, N = 1134	Known MASLD	Suspected MASLD	Cryptogenic cirrhosis
		N = 521	N = 497	N = 116
Blood tests	88.6% (1005)	89% (465)	89% (443)	84% (97)
Imaging	59.5% (675)	48% (248)	68% (338)	77% (89)
Serum fibrosis markers	26.9% (304)	30% (155)	24% (119)	26% (30)
Elastography^a^	41.2% (467)	45% (233)	43% (215)	16.4% (19)
Liver biopsy	14.3% (162)	12.5% (65)	17% (85)	10% (12)
None	4.8% (54)	4.6% (24)	4.6% (23)	6% (7)

a By vibration-controlled transient elastography. Percentage counts exceed 100% because of multiple procedures scheduled in same patient.

We next specifically studied the prescription of fibrosis non-invasive tests in the whole population of 1134 patients during the initial visit. Non-invasive fibrosis tests were not prescribed at all in 558 pts (49%), elastography was prescribed alone in 272 (24%), elastography with a proprietary serum fibrosis marker in 195 (17%) and a proprietary serum fibrosis marker alone in 109 (10%). Elastography was mainly scheduled by VCTE with only a marginal number (N = 6) by shear-wave elastography.

### Overall assessment at the end of follow-up

#### Follow-up procedures

A follow-up visit aimed at evaluating the results of scheduled procedures and establishing a diagnosis and its severity was conducted in all patients, while 243 patients had 2 visits of follow-up and 138 patients had three visits. Thus, the mean number of follow-up visits per included patient was 1.46 (s.d. 0.70).

The additional follow-up visits led, in some patients, to the prescription of additional procedures, mostly based on results generated by the initial set of prescribed procedures. In the end, 81.7% of patients had blood tests to explore metabolic dysfunction (glycemia, lipid panel, or fasting insulin), 44% had additional blood tests to exclude other chronic liver diseases, 66.4% had hepatic imaging (ultrasound or CT scan or MRI) and 20.4% had a liver biopsy performed. As far as non-invasive assessments for fibrosis, 47.2% had elastography and 37.3% had serum fibrosis biomarkers performed. While 19.1% had both performed, 28% had only elastography, 18.2% only serum biomarkers and 34.7% had neither performed. The breakdown per country is shown in Supplementary Figure S3.

#### Final diagnosis

At the end-of-study visit the PP established a final diagnosis which was based on the results of the liver biopsy in 231 patients (histological diagnosis) or on an overall assessment of available data collected since the baseline visit in the 903 pts who did not have a liver biopsy performed (clinical diagnosis) (Fig. 3). In the 231 patients with histological examination the diagnosis was steatohepatitis 53%, steatosis without steatohepatitis 18%, cryptogenic cirrhosis 8% and other (including no MASLD, non-specific lesions, normal liver) 20%. Combining these two assessments, 69% of patients had a MASLD diagnosis confirmed, 11.2% had a diagnosis of cryptogenic cirrhosis (including burned out MASH *and cryptogenic verum*) and 19.8% had a final diagnosis other than MASLD or cryptogenic cirrhosis (including undetermined, unknown etc, Supplementary Table S2). Baseline characteristics associated with a MASLD diagnosis are shown in Supplementary Table S3.

**Fig. 3:**
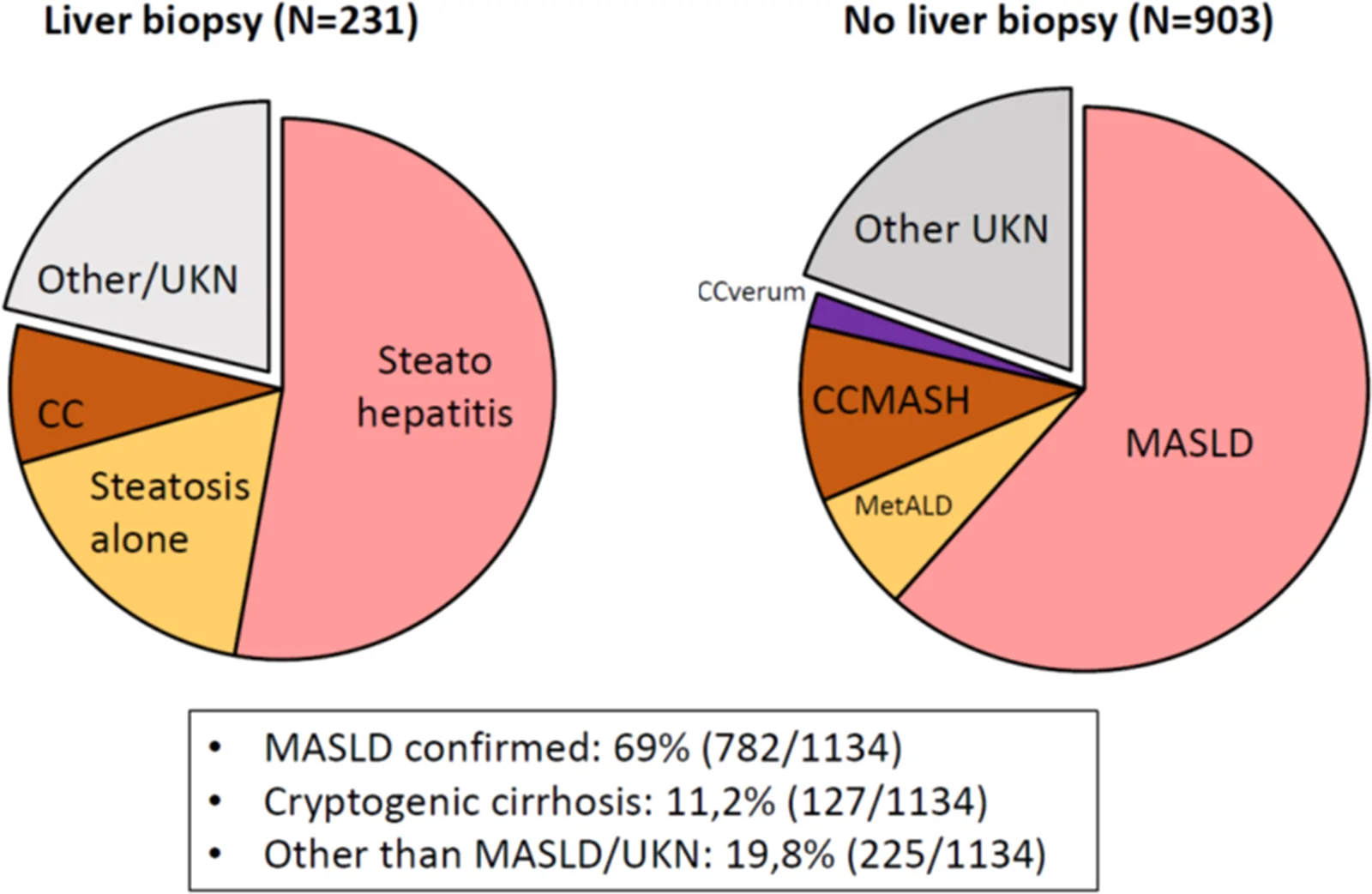
Final, adjudicated diagnostic categories. The diagnostic categories were established based on histological criteria in those who had a liver biopsy performed, and on clinical criteria in those without a liver biopsy. The box states the overall diagnostic categories based on all available information. *UKN, unknown*.

#### Severity of the liver condition

Similarly, the suspected severity of the liver condition was assessed based on the fibrosis stage in the 231 patients explored by liver biopsy or on an overall assessment of elastography data, clinical and biological data and historical data (in patients already followed) in the 903 patients who did not have a liver biopsy (Fig. 4). Fibrosis stages (NASH CRN) in patients with a liver biopsy performed were: stage 0: 14%, stage 1, 25%, stage 2, 25%, stage 3 17% and cirrhosis 20%. Combining these two approaches and adjudicating occasional cases of discordant results between biopsy and elastography resulted in the following severity assessment: 37.9% of patients had no or mild hepatic fibrosis, 22.7% had significant fibrosis (equivalent to stages 2 and 3), 21.3% had cirrhosis while in 18.2% the fibrosis assessment could not be determined. Baseline characteristics associated with a MASLD diagnosis are shown in Supplementary Table S4.

**Fig. 4:**
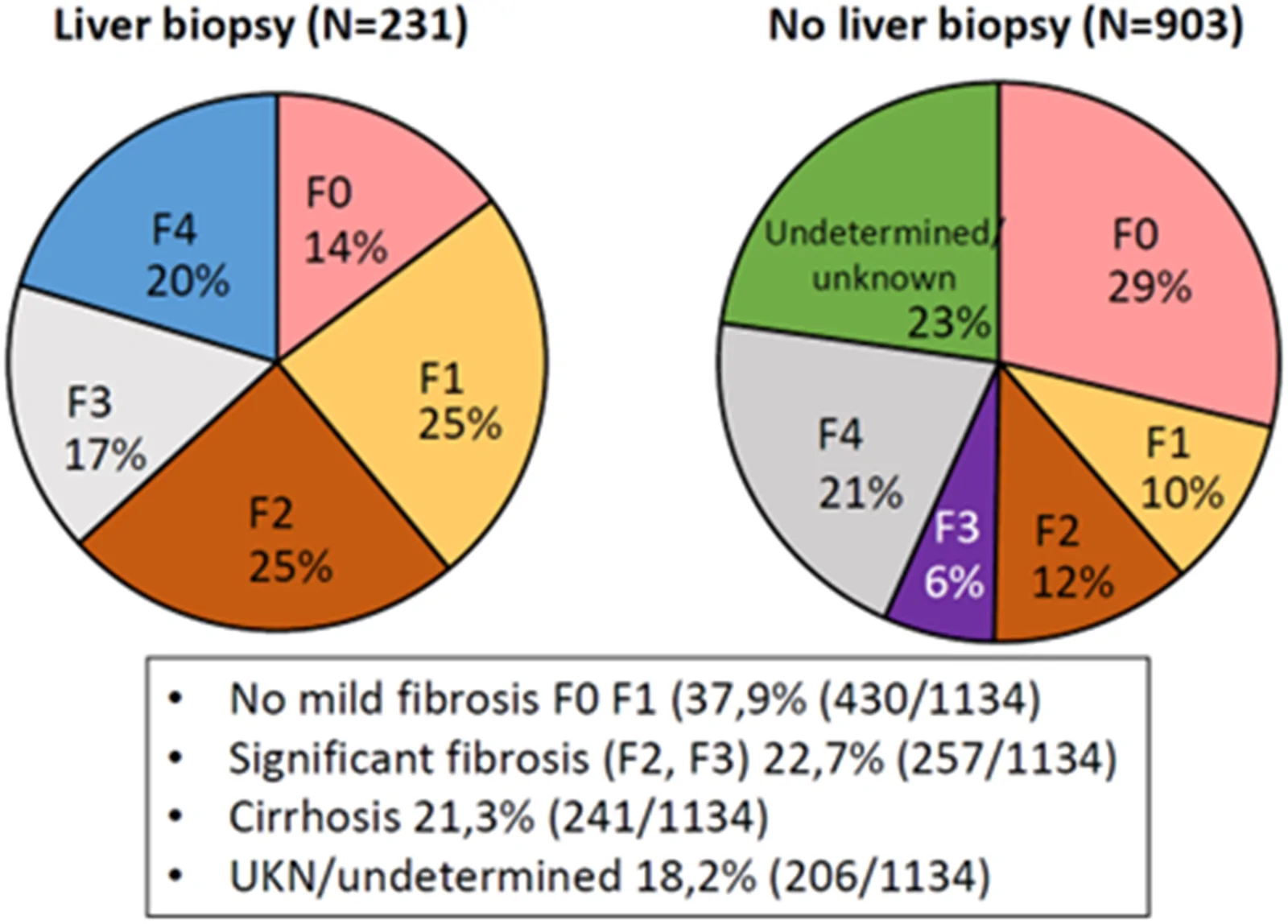
Final, adjudicated fibrosis severity. The prevalence of histologically defined fibrosis stages in patients who had a liver biopsy performed, in those with only non-invasive tests performed and in the whole cohort (box), taking into account all available information. *UKN, unknown*.

#### Further referrals

Based on results of the different procedures and the overall clinical assessment, 144 patients with MASLD or cryptogenic cirrhosis (15.8%) were referred to another specialist in the cardio-metabolic field: most referrals were for a nutritionist/dietician (N = 84), endocrinologist (N = 59) cardiologist (N = 42) and nephrologist (N = 5).

### Cost analysis

Table 2 shows an estimate of the cost incurred by the initial visit, follow-up visits, diagnostic procedures performed and visits for specialist referral with a breakdown by country. The overall cost per patient consulting for diagnosed or suspected MASLD, in the participating center, focuses on investigations and excludes cost of therapeutics that may or may not have been prescribed or followed by patients. It ranges from 95€ to 695€ depending on the country, local practices and specifics of the health care system. Overall costs were highly variable between countries (Supplementary Figure S4). In multivariable mixed-effects models accounting for center-level clustering, higher costs were independently associated with several patient characteristics. Each unit increase in BMI was associated with a 1.7% increase in cost (cost ratio 1.017, 95% CI 1.008–1.026; p < 0.001). Patients with diabetes had 17% higher costs compared to non-diabetic patients (cost ratio 1.17, 95% CI 1.05–1.31; p = 0.006). Dyslipidaemia was also associated with increased costs (cost ratio 1.13, 95% CI 1.01–1.26; p = 0.031). New patients incurred substantially higher costs compared to follow-up visits, with an estimated 25% increase (cost ratio 1.25, 95% CI 1.12–1.40; p < 0.001). In contrast, age, sex, and arterial hypertension were not significantly associated with cost after adjustment (Supplementary Table S5).

**Table 2:** Cost incurred by medical visits and procedures performed in patients with known or suspected MASLD.

	Germany N = 297	UK N = 142	France N = 247	Switzerland N = 147	Italy N = 177	Spain N = 60	Slovakia N = 64
**Medical visits**							
Initial	297	142	247	147	177	60	64
Follow-up	463	179	431	173	192	137	78
Total cost	137,511	25,418	106,457	25,431	33,984	8220	4992
Cost per patient	128	201	189	133	54	49	26
**VCTE**							
Performed	110	36	131	129	100	7	22
Total cost	15,730	5652	4061	15,867	4500	525	660
Cost per patient	53	40	16	108	25	9	10
**Liver biopsy**							
Performed	113	8	36	22	51	0	1
Total cost	114,356	4664	49,644	19,030	19,074	0	578
Cost per patient	385	33	201	129	108	0	9
**Ultrasound**							
Performed	244	56	67	92	77	55	43
Total cost	17,812	4536	3618	12,972	4697	2035	1290
Cost per patient	60	32	15	88	27	34	20
**Other imaging procedures**							
CT scan/MRI Performed	15/16	7/5	10/20	12/14	7/4	2/1	1/5
Total cost	4025	2915	4025	20,473	1356	562	957
Cost per patient	14	21	16	139	8	9	15
**Blood tests**^a^							
Total cost	8890	4651	7826	9724	3813	1886	765
Cost per patient	30	33	32	66	22	31	12
**Specialist referral**							
Number of referrals	2	20	79	31	48	3	7
Total cost	160	2800	5530	4650	2880	150	210
Cost per patient	0.50	20	22	32	16	2.5	3
**Overall cost per patient**	671	380	491	695	260	135	95

VCTE, vibration-controlled transient elastography.

a Includes any of: liver enzymes (AST, ALT, Gamma GT, alkaline phosphatase) glycemia, serum insulin, lipid panel.

As a comparator, the estimated price for the initial work-up of a patient with active HCV infection is shown in Table 3 and the ratio between expenses generated by the referral of a patient with MASLD or suspected MASLD and the ones generated by the referral of a patient with suspected or confirmed HCV infection is shown in Fig. 5. The cost incurred by a MASLD diagnosis was higher than that of an HCV diagnosis in Germany and France (35% and 28% higher, respectively), similar in Switzerland and lower in United Kingdom, Italy, Spain and Slovakia (Table 3). When taking into account additional expenses related to the decision to treat HCV infection and check the viral response (cost of antiviral medication excluded) the cost of managing a patient with MASLD were uniformly lower than those of managing a patient with HCV (Table 3 and Fig. 5).

**Table 3:** Estimated real costs (euros) for MASLD management vs theoretical cost for management of chronic hepatitis C.

	Germany	UK	France	Switzerland	Italy	Spain	Slovakia
Overall cost per MASLD patient	671	380	491	695	260	135	95
Overall cost HCV before treatment	496	709	384	723	362	357	237
Overall cost HCV after treatment	776	1167	702	1149	594	567	381
Ratio^a^ before HCV treatment	1.35	0.54	1.28	0.96	0.72	0.38	0.40
Ratio^a^ after HCV treatment	0.86	0.33	0.70	0.60	0.44	0.24	0.25

HCV, chronic hepatitis C.

a Refers to the ratio between MASLD and HCV costs.

**Fig. 5:**
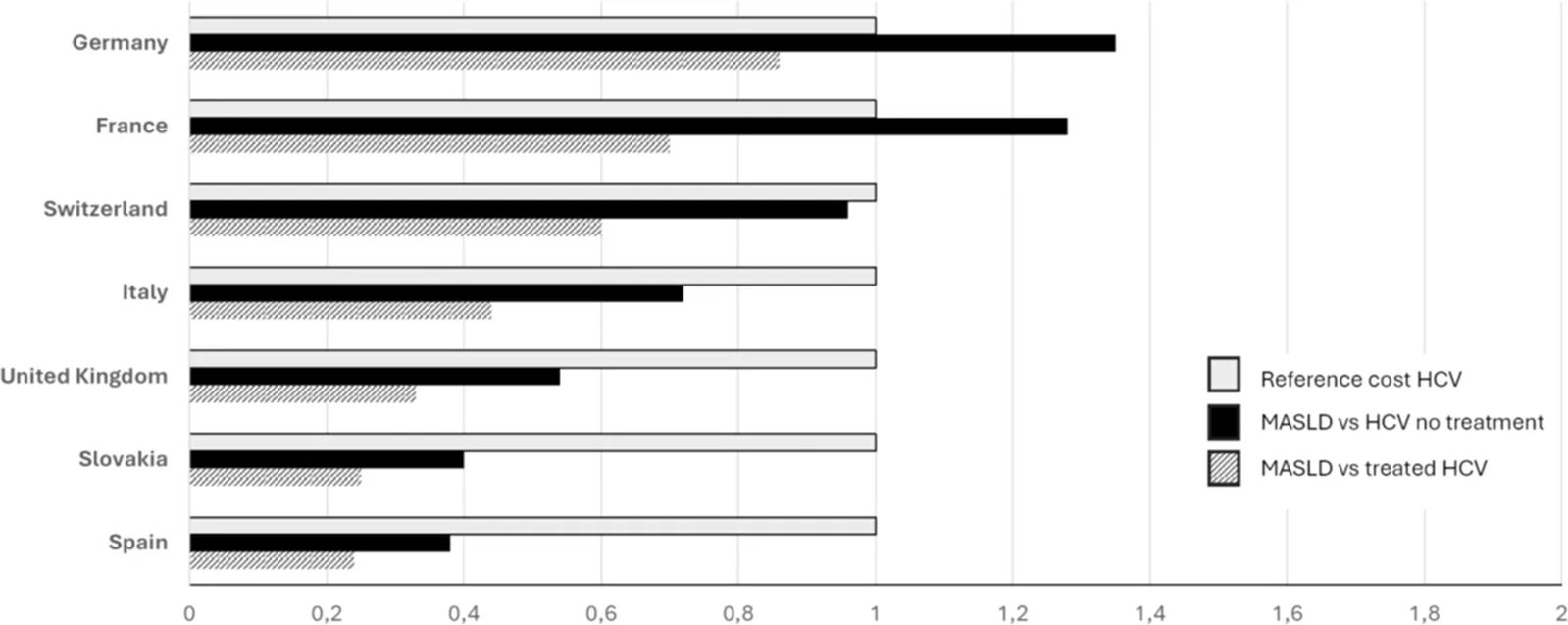
The ratio between the estimated cost for MASLD patients and the theoretical cost for managing hepatitis C taken as reference (gray boxes). Cost for MASD vs cost for hepatitis C before HCV antiviral treatment (black boxes) and vs cost for hepatitis C including antiviral treatment (hatched boxes).

## Discussion

This prospective, international, multicentre study provides the first real-world evidence of the burden of MASLD in clinical practice in tertiary care hepatogastroenterology centers from different European countries. A quarter of hepatological visits are related to patients with MASLD, whether already diagnosed or suspected, and this represents, overall, the second, single most common cause for a specialized visit, after viral hepatitis. In several countries such as France, Italy, Spain or Slovakia, the proportion is even higher (29–43%). After extensive evaluation and follow-up visits, the diagnosis of MASLD was confirmed in a large majority (80%) of cases. The fibrotic severity was particularly high, as 44% of patients had significant fibrosis (equivalent to histological stage 2 or higher) and 29% advanced fibrosis (equivalent to stage 3 or higher). A sizeable proportion of patients present with cryptogenic cirrhosis (11%) the majority of which were due to burnt-out MASH given current or prior exposure to metabolic risk factors. The outpatient clinic setting is important since the large majority of MASLD patients is asymptomatic with few patients necessitating hospitalization or surgical procedures including liver transplantation. Thus, data ignoring patients seen in this setting will most probably underestimate the bulk of medical expenses and resources truly allocated to MASLD. The high prevalence documented here forecasts a surge in severe cases to be expected in the future. While in the United States MASLD is among the top two causes of end stage liver disease and hepatocellular cancer listed for liver transplantation,^15^ in Europe it is a rather marginal cause, albeit currently increasing in incidence.^16^ The increasing incidence in children and adolescents will also add to the healthcare burden,^17^^,^^18^ as an earlier exposure to obesity and diabetes will most probably result in the occurrence of the most severe stages of the disease at an earlier age.^19^

The 25% prevalence of MASLD among patients seeking specialized consultations documented in this real-world study, with multimodal, specialist-adjudicated diagnostic procedures will help inform future studies on the health-care burden of MASLD. Multiple prevalence estimates from different diagnostic contexts exist: some rely on modeling using estimates for obesity or diabetes in the general population instead of a direct diagnosis of MASLD^5^^,^^6^; others rely on data extracted from electronic medical records largely prone to coding errors^20^; yet others are based on health-care registries which included only biopsied patients.^7^ These diverse settings may result in highly discordant estimates^20^, ^21^, ^22^, ^23^ without specifically answering the question of the real burden of MASLD actually seen in specialist clinical practice. The latter question is relevant as diverse referral pathways are now being recommended by many scientific societies^11^, ^12^, ^13^, ^14^ which, if implemented, may only add to the current burden of care. By using a prospective, rigorous, specialist-adjudicated diagnosis, as per real clinical practice, we estimated the prevalence and disease severity of MASLD and identified the diagnostic procedures performed thus providing a more precise estimate in the context of different European countries and within their particular health-care systems.

The design of the current study is unique as it uses individual patient data collected prospectively in routine clinical practice and adjudicated by specialist hepatogastroenterologists during both first and follow-up visits. It allows to estimate the proportion of patients seen for MASLD vs other diseases and the consequent use of healthcare resources. Data on patients with MASLD investigated in the clinic have been reported by Geier et al. from a nationwide registry in Germany,^24^ by Ampuero et al. from the Spanish Hepamet Registry^25^ while a similar nationwide registry is ongoing in Italy.^26^ However, all these studies included only patients with an established diagnosis of MASLD thus not allowing the evaluation of the proportion of MASLD among other reasons for specialized visits, a major objective of the current analysis and an important indicator of the health care burden of MASLD in outpatient visits. One other study used data derived from real patients following regular consults.^9^^,^^27^ However, the MASLD patient cases were selected by the surveyed physicians, thus introducing the possibility of recall bias as suggested by the exceedingly high proportion of 66% of patients reported to undergo liver biopsy.^9^ Additionally, information on the larger denominator from which these cases were extracted is lacking. Moreover, the lack of longitudinal data also limited the full description of medical resources only to those prescribed at the inclusion visit.

Data on actual health care resource utilization are equally scarce. This study shows that most MASLD patients seen in tertiary care centers will undergo a large variety of blood tests while a majority of them will have imaging methods performed. Surprisingly, liver biopsy has not quite disappeared from clinical practice, since around 20% of patients still had a liver biopsy performed. Particularly striking in this study is the limited use of non-invasive methods for fibrosis staging. Less than half of patients had an elastography performed, a third had serum NITs and only 19% had both. In fact, a third of patients did not have any non-invasive investigation of fibrosis stage with no notable differences between first-time (suspected MASLD) and already known MASLD. This low level of uptake is surprising as an assessment of fibrosis burden is recommended both at diagnosis and during follow-up for disease monitoring.^11^^,^^13^^,^^14^^,^^28^^,^^29^ Local practice differs between European centers with striking disparities in the use of serum biomarkers with some centers rarely, if ever, using direct collagen biomarkers (Switzerland, UK, Spain) which is unexpected given the existence of common European guidelines.^30^ Other studies have shown that even simple, non-proprietary serum biomarkers are not being performed before specialist referral.^3^ Taken together, these data suggest that much progress still needs to be made before a comprehensive non-invasive fibrosis staging be optimally applied for patients with MASLD. Since this study was conducted, however, several reports have shown the prognostic value of non-invasive biomarkers^31^, ^32^, ^33^ in addition to their diagnostic value,^34^ and, pending reimbursement, it is probable that their use in the future will increase. Conversely, it is encouraging that the work-up included the search for cardiometabolic risk factors in MASLD patients seen for the liver condition. Thus, practicing hepatologists, from all seven countries, recognize the association of MASLD with extra-hepatic diseases related to the underlying metabolic dysfunction. Nonetheless, the proportion of patients referred to another specialist was low, around 16%. Thus, even in specialized clinical practice, the implementation of a truly holistic management of MASLD patients’ needs further optimization.

Since all visits and diagnostic procedures were carefully recorded, we were able to estimate the cost related to MASLD outpatient management. In order to account for the striking disparities in health-care costs between European countries, we compared the costs of medical visits and procedures performed for patients with MASLD with those incurred by a diagnosis of chronic hepatitis C. In Germany, France and Switzerland the cost of investigating MASLD exceeded or was equal to that of chronic HCV before the initiation of antiviral therapy. Thus, the financial burden of exploring and monitoring patients with steatotic liver disease is, at least in some European countries, within the range of the initial work-up of chronic hepatitis C. Accounting for the additional procedures necessary for monitoring treatment response and viral eradication in chronic hepatitis C exceeded the cost of exploring MASLD patients. However, this study was performed before the approval of any specific therapy for MASLD. In September 2025 EMA has granted a conditional marketing authorization for resmetirom and thus it is expected that once MASH therapies become available, the direct cost of managing MASLD patients will further increase. Since MASH treatments are not curative and will be administered long-term, this cost will most probably far exceed, during a patient's lifetime, that of managing chronic hepatitis C.

Available data on health care cost for MASLD are either based on prevalence modeling,^35^ on administrative claims databases prone to incomplete or erroneous coding^36^ or on patients selected by surveyed physicians^27^ with the limitations of recall bias noted above. Within these limitations, the rare available data from Europe suggest that the total annual mean cost of diagnostic tests and procedures was 210 € in France and 187 € in Germany while the cost related to hospitalization and medical visits was 840 € in France and 303 € in Germany.^27^ While hospitalization and intensive care unit visits are rarely if ever necessary in uncomplicated MASLD, which is by definition the patient population seen in outpatient visits, these estimates are insufficiently precise to describe the real costs incurred by the segment of the population studied in the current work. By strictly selecting a homogenous population of outpatients with MASLD and precisely identifying visits and procedures actually performed, the current study provides useful and more accurate estimates of costs incurred by initial management of these patients. The health-care burden observed in this study is factual but does not necessarily predict future trends. On one hand, a more widespread use of biomarker-based risk stratification strategies in primary or secondary care might reduce specialist referral for many low-risk patients with associated reductions in cost and work burden for specialists. On the other hand, in some patients in the current study, the use of non-invasive tests or the exploration of comorbidities including referrals to other specialists was suboptimal. Improving on this aspect will increase associated costs and workload for specialists. In any case the identification of the current state of practice is necessary for a better future allocation of health-care ressources.

This study has several strengths. It was conducted prospectively in outpatient clinics, included consecutive patients in real-world conditions and analyzed individual patient data. The prespecified one year follow-up allowed to capture all diagnostic procedures and additional visits that were necessary before establishing a firm diagnosis (in case of suspected MASLD) and the severity of liver injury. Most importantly the diagnosis and severity of the disease were adjudicated by practicing hepatologists, on an individual basis, from real-patient data. A central oversight of collected data verified the final estimate of disease severity in each patient, based on a combination of invasive (liver biopsy) and non-invasive patient-level data with adjudication of occasional discrepancies. Overall, this reflects as close as possible the everyday practice in specialized hepatology centers. Thus, both diagnosis and severity of the disease were determined to the best of our specialized knowledge.

Alongside the above-mentioned strengths, the current study has several limitations, one of which was that it was performed in highly specialized tertiary care centers. Although specific hepatological investigations for MASLD are, by-and-large, performed in specialized centers, this study may not reflect practices and patient profiles occasionally investigated outside tertiary centers, which still impact on the overall health-care burden of MASLD. The principal investigators of the eight participating centers have a vested interest in MASLD care and research, however each center included several other investigators with diversified hepatological practice. While it is possible that, to a certain extent, differences in management between centers may be subject to pre-determined pathways proper to each center, this was not specifically tested in the present study. Accurate and complete cost estimation is challenging, mainly due to variability in listed prices, incomplete reporting, unclear inclusion of supplies and bundled services, hidden costs and out of pocket expenses for patients.^37^, ^38^, ^39^ Besides, there is significant price variation across hospitals and procedures within a single country,^40^ therefore the costs estimated here may not be considered definitive or representative of entire countries. Finally, societal costs, including indirect costs, were not assessable in this study. Future large and multicentric studies using real world individual patient data, as in the current work, should comprehensively quantify all aspects of the health-care burden of MASLD. In particular, variability between center and country practices should be accounted for, as well as monitoring on specfic pharmacotherapy, once these drugs become largely available in this indication.

In conclusion, this multicentric, prospective, real-world practice study based on expert adjudication of patient-level data has shown that MASLD is now the second most common cause for outpatient visits in hepatology centers in seven European countries. Although mostly asymptomatic, advanced fibrosis was present in around a third of patients while one out of ten having cryptogenic cirrhosis attributable to advanced (burnt-out) MASLD. This generated substantial medical investigations whose overall cost equaled that of the exploration of chronic hepatitis C. The use of non-invasive tests was still suboptimal and a multidisciplinary care insufficiently implemented. This study provides real practice estimates of the health-care burden and utilization of diagnostic procedures that are needed for a rational implementation of health-care resources in an era of increasing prevalence of MASLD.

## Contributors

Conceptualization and study design: VR; Input into study design: JFD, RP, EB, FM, MRG, QMA, JS; data acquisition: all authors; data analysis: VR; statistical analysis: MP and VR; data curation LK; writing of the manuscript: VR; revision of the manuscript: all authors; All authors read and approved the final version of the manuscript, and were responsible for the decision to submit it for publication. Access and verification of final data: VR, MP, LK; Submission to the Journal: VR.

## Data sharing statement

De-identified individual participant data supporting the findings of this study will be made available from the corresponding author upon reasonable request.

## Declaration of interests

None that are relevant to this study, for all authors.

## Figures and Tables

**Supplementary Figure S1:**
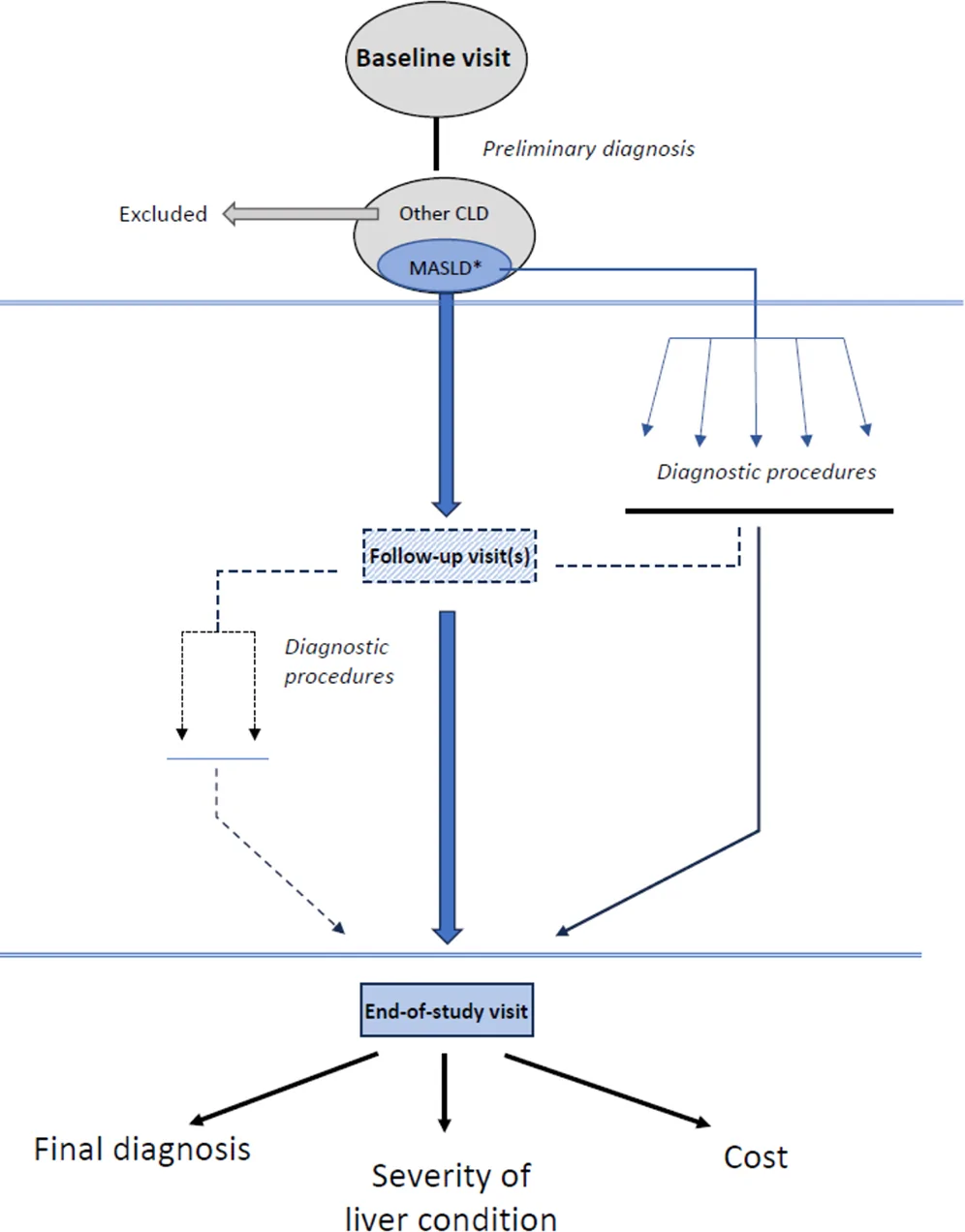
Study design.

**Supplementary Figure S2:**
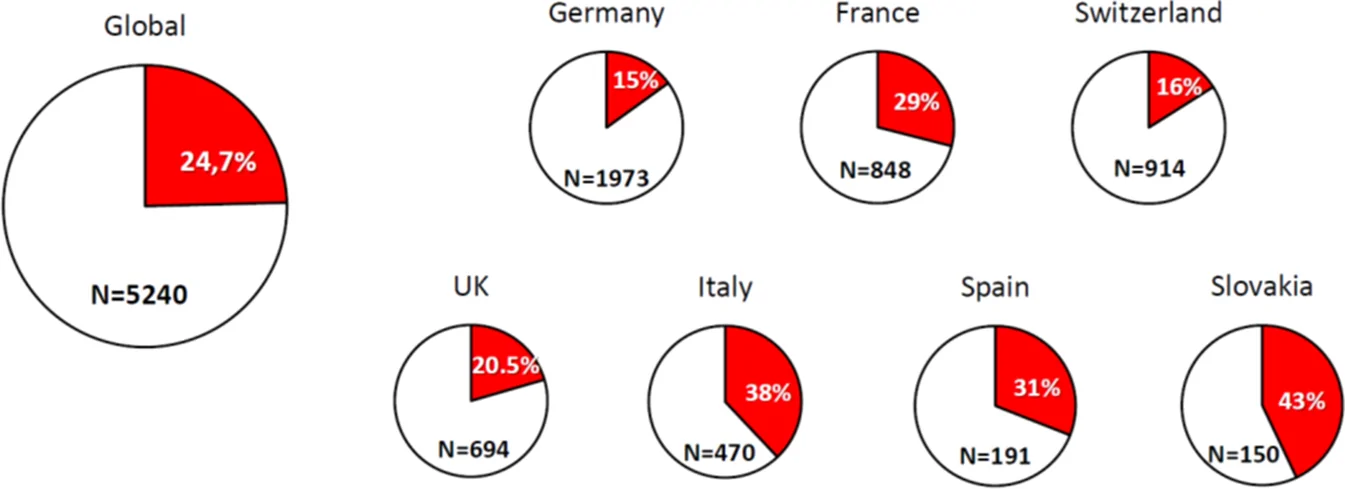
Proportion of patients with a diagnosis of MASLD or suspected MASLD at the baseline visit.

**Supplementary Figure S3:**
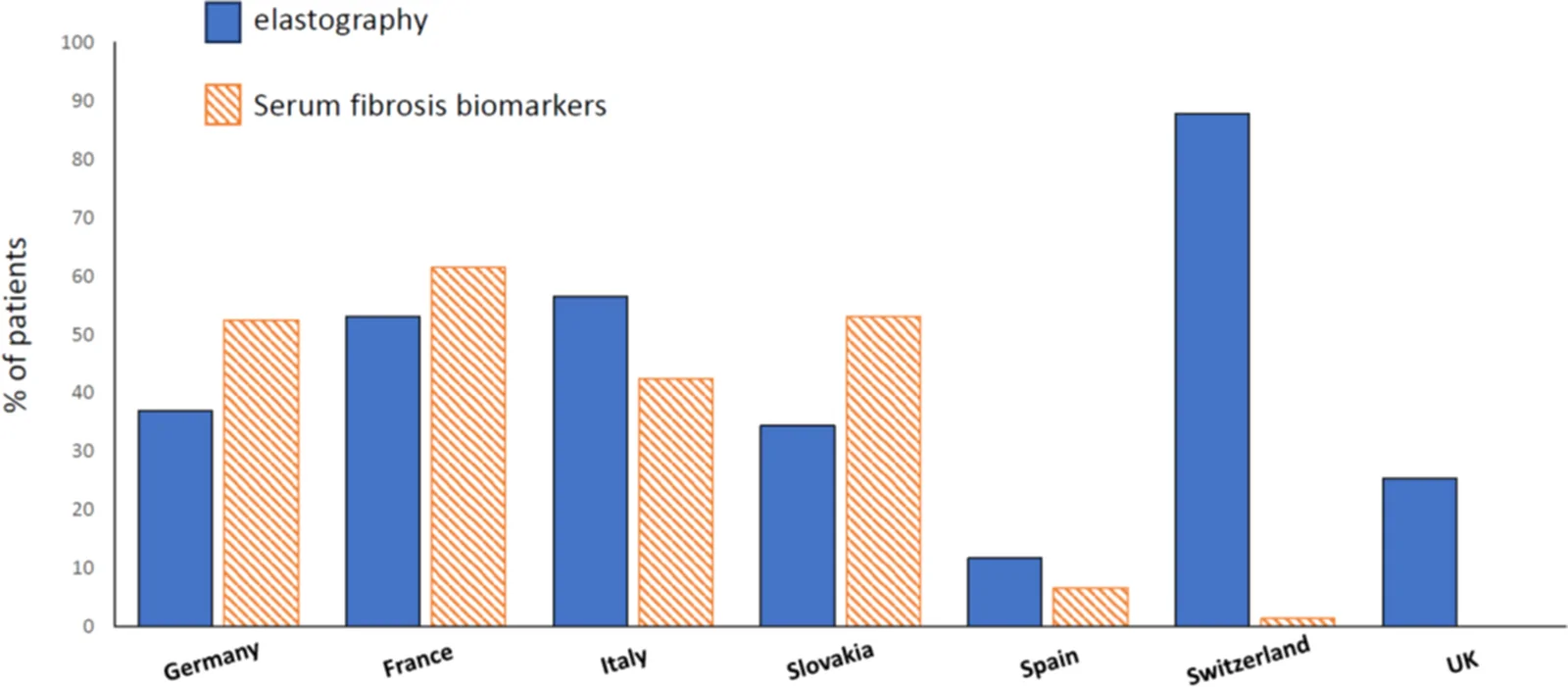
Proportion of patients who performed elastography (by VCTE) and serum fibrosis biomarkers. (VCTE: vibration controlled transient elastography).

**Supplementary Figure S4:**
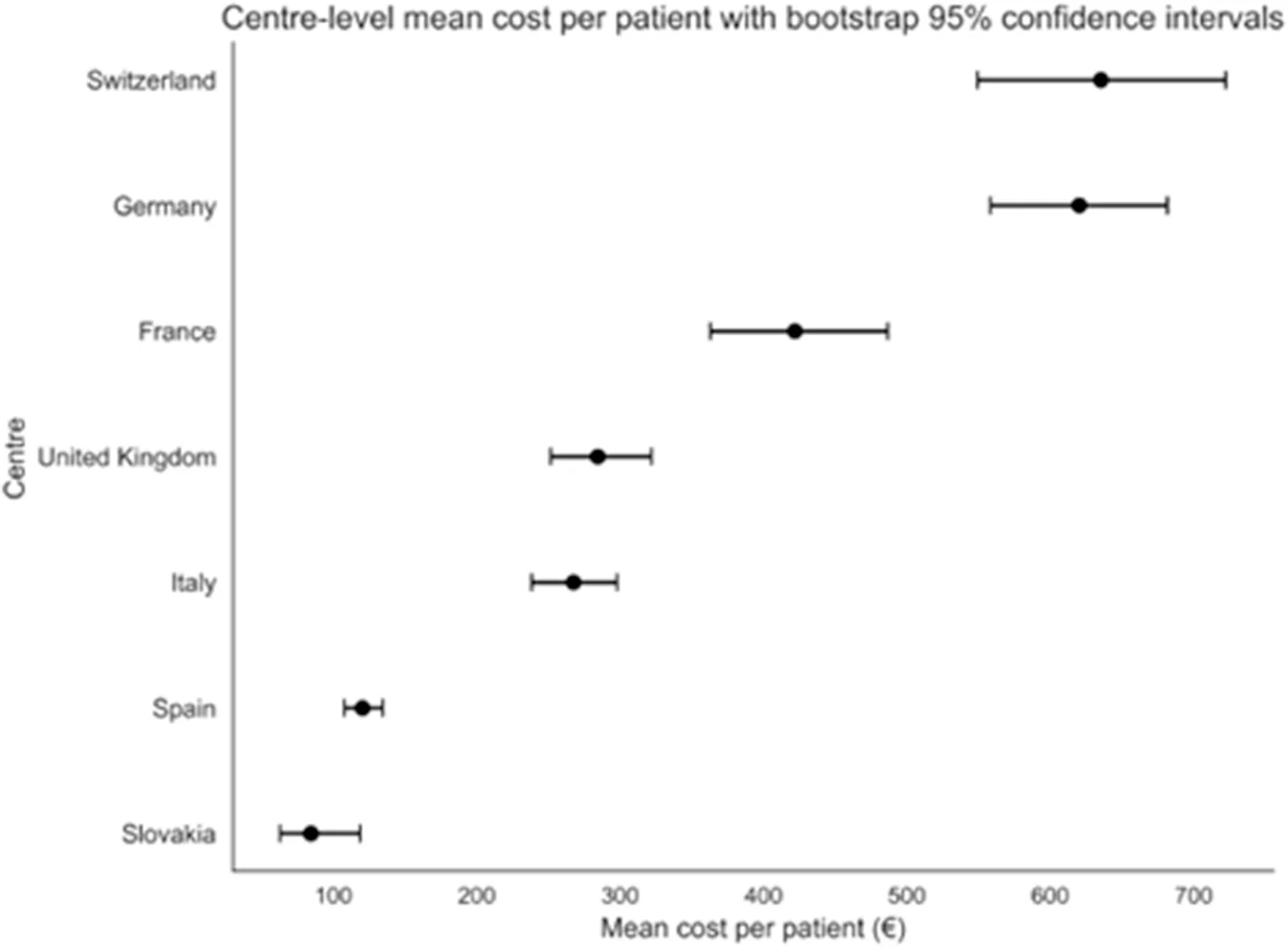
Random effects analysis for the centre level mean cost per patient (with bootstrap 95% confidence analysis).

## Appendix A Supplementary data

Supplementary Table S1 Characteristics of the 1134 known or suspected MASLD patients included for follow-up.

Supplementary Table S2 Other diagnoses (N=225) within the 1134 patients with follow-up.

Supplementary Table S3 Comparison of complete-case and multiple imputation (MICE) analyses for determinants of MASLD-related diagnosis.

Supplementary Table S4 Comparison of complete-case and multiple imputation (MICE) analyses for determinants of severe liver disease among patients with MASLD-related diagnosis.

Supplementary Table S5 Determinants of overall cost per patient, including procedures and referrals, from multivariable mixed-effects models accounting for center-level clustering.
