# Supplementary material for: Health-care burden of metabolic dysfunction-associated steatotic liver disease in European tertiary-care outpatient clinics (CONSTANS): a prospective multicentre cohort study
Source: Lancet Reg Health Eur. 2026 Oct 29;71:101893. doi: 10.1016/j.lanepe.2026.101893 (PMC13743415; doi:10.1016/j.lanepe.2026.101893)
Supplement: Supplementary file 1 [file mmc1.docx]

**Supplementary Tables.**

**Supplementary Table 1.** Characteristics of the 1134 known or suspected MASLD patients included for follow-up.

| Age, mean (s.d.), yrs | 56.9 (13.9) |
| --- | --- |
| Male sex, % | 52 |
| BMI, mean (s.d.), kg/m² | 30.4 (6.2) |
| Comorbidities |  |
| Type 2 diabetes, % | 34.3 |
| Arterial hypertension, % | 46.9 |
| Dyslipidemia, % | 58.3 |
| Obstructive sleep apnea, % | 8.5 |
| Alcohol consumption, % |  |
| None | 71.4 |
| <30 g/day* | 24.7 |
| 30-50 g/day | 3.2 |
| >50 g/day | 0.7 |
|  |  |
|  |  |
|  |  |
