# Supplementary material for: Health-care burden of metabolic dysfunction-associated steatotic liver disease in European tertiary-care outpatient clinics (CONSTANS): a prospective multicentre cohort study
Source: Lancet Reg Health Eur. 2026 Oct 29;71:101893. doi: 10.1016/j.lanepe.2026.101893 (PMC13743415; doi:10.1016/j.lanepe.2026.101893)
Supplement: Supplementary file 2 [file mmc2.docx]

**Supplementary Tables.**

**Supplementary Table 2**. Other diagnoses (N=225) within the 1134 patients with follow-up.

| Condition | N |
| --- | --- |
| Neoplasia | 29 |
| Autoimmune (incl. suspicion of autoimmune hepatitis, PBC, PSC, overlap syndromes) | 23 |
| Suspicion of drug-induced liver injury | 16 |
| Viral origin (incl. HSV, HBV, HCV, HEV) | 12 |
| Alcohol-related liver disease | 14 |
| Genetic diseases (incl. HFE-related hemochromatosis, alpha1 antitrypsin deficiency, genetic cholestasis, Apolipoprotein B deficiency) | 9 |
| Vascular disorders (incl. cardiac insufficiency, porto-sinusoidal vascular disease) | 13 |
| Miscellaneous | 17 |
| Unknown/undetermined | 92 |
