# Supplementary material for: Health-care burden of metabolic dysfunction-associated steatotic liver disease in European tertiary-care outpatient clinics (CONSTANS): a prospective multicentre cohort study
Source: Lancet Reg Health Eur. 2026 Oct 29;71:101893. doi: 10.1016/j.lanepe.2026.101893 (PMC13743415; doi:10.1016/j.lanepe.2026.101893)
Supplement: Supplementary file 3 [file mmc3.docx]

**Supplementary Tables.**

**Supplementary Table 3.** Comparison of complete-case and multiple imputation (MICE) analyses for determinants of MASLD-related diagnosis.

| **Variable** | **Complete-case OR (95% CI)** | **MICE OR (95% CI)** |
| --- | --- | --- |
| Age (per year) | 1.01 (0.99–1.02) | 1.01 (0.99–1.02) |
| Female sex | 0.70 (0.41–1.21) | 0.77 (0.53–1.12) |
| BMI (per kg/m²) | 1.20 (1.12–1.30) | 1.19 (1.12–1.27) |
| Type 2 diabetes | 3.84 (2.36–6.23) | 2.96 (1.97–4.44) |
| Arterial hypertension | 1.07 (0.70–1.63) | 1.12 (0.76–1.65) |
| Dyslipidaemia | 1.56 (0.92–2.67) | 1.30 (0.88–1.93) |
| New patient | 0.46 (0.25–0.84) | 0.50 (0.30–0.82) |

MICE: multiple imputation by chained equations. Sensitivity analyses to account for missing data were performed using MICE: results obtained after multiple imputation were highly consistent with the primary complete-case analyses.
