# Supplementary material for: Health-care burden of metabolic dysfunction-associated steatotic liver disease in European tertiary-care outpatient clinics (CONSTANS): a prospective multicentre cohort study
Source: Lancet Reg Health Eur. 2026 Oct 29;71:101893. doi: 10.1016/j.lanepe.2026.101893 (PMC13743415; doi:10.1016/j.lanepe.2026.101893)
Supplement: Supplementary file 4 [file mmc4.docx]

**Supplementary Tables.**

**Supplementary Table 4.** Comparison of complete-case and multiple imputation (MICE) analyses for determinants of severe liver disease among patients with MASLD-related diagnosis

| **Variable** | **Complete-case OR (95% CI)** | **MICE OR (95% CI)** |
| --- | --- | --- |
| Age (per year) | 1.03 (1.02–1.04) | 1.03 (1.02–1.04) |
| Female sex | 0.80 (0.52–1.23) | 0.90 (0.67–1.22) |
| BMI (per kg/m²) | 1.04 (1.03–1.06) | 1.04 (1.02–1.06) |
| Type 2 diabetes | 1.82 (1.29–2.57) | 1.97 (1.45–2.69) |
| Arterial hypertension | 1.78 (1.31–2.42) | 1.64 (1.23–2.19) |
| Dyslipidaemia | 1.00 (0.65–1.34) | 0.88 (0.67–1.17) |
| New patient | 1.26 (0.86–1.85) | 1.03 (0.65–1.33) |

MICE: multiple imputation by chained equations. Sensitivity analyses to account for missing data were performed using MICE: results obtained after multiple imputation were highly consistent with the primary complete-case analyses.
