# Supplementary material for: Health-care burden of metabolic dysfunction-associated steatotic liver disease in European tertiary-care outpatient clinics (CONSTANS): a prospective multicentre cohort study
Source: Lancet Reg Health Eur. 2026 Oct 29;71:101893. doi: 10.1016/j.lanepe.2026.101893 (PMC13743415; doi:10.1016/j.lanepe.2026.101893)
Supplement: Supplementary file 5 [file mmc5.docx]

**Supplementary Tables.**

**Supplementary Table 5:** Determinants of overall cost per patient, including procedures and referrals, from multivariable mixed-effects models accounting for center-level clustering.

| **Variable** | **Cost ratio** | **95% CI** | **p-value** |
| --- | --- | --- | --- |
| Age (per year) | 1.00 | 0.99-1.00 | 0.187 |
| Female sex | 0.96 | 0.87-1.07 | 0.461 |
| BMI (per kg/m²) | 1.02 | 1.01-1.03 | <0.001 |
| Type 2 diabetes | 1.16 | 1.04-1.30 | 0.009 |
| Arterial hypertension | 1.08 | 0.96-1.21 | 0.196 |
| Dyslipidemia | 1.11 | 0.99-1.24 | 0.062 |
| New patient | 1.25 | 1.12-1.40 | <0.001 |
